# Supplementary material for: A Critical View of the Application of the APEX Software (Aqueous Photochemistry of Environmentally-Occurring Xenobiotics) to Predict Photoreaction Kinetics in Surface Freshwaters
Source: Molecules. 2019 Dec 18;25(1):9. doi: 10.3390/molecules25010009 (PMC7017383; doi:10.3390/molecules25010009)
Supplement: Supplementary file 1 [file molecules-25-00009-s001.zip › 3.2.4_gcc-4.4.0.zip!/3.2.4_gcc-4.4.0/3.2.4_gcc-4.4.0/doc/octave/html/interpreter/Function-Index.html]

Function Index - Untitled


Next: Operator Index,
Previous: Concept Index,
Up: Top


---

## Function Index

- `(`: Command Line Options
- `(`: Controlling Subprocesses
- `-all`: Getting Help
- `=`: Sparse Linear Algebra
- `=`: Nonlinear Equations
- `=`: Sparse Linear Algebra
- `abs`: Complex Arithmetic
- `accumarray`: Sums and Products
- `acos`: Trigonometry
- `acosd`: Trigonometry
- `acosh`: Trigonometry
- `acot`: Trigonometry
- `acotd`: Trigonometry
- `acoth`: Trigonometry
- `acsc`: Trigonometry
- `acscd`: Trigonometry
- `acsch`: Trigonometry
- `addlistener`: Object Groups
- `addpath`: Manipulating the load path
- `addproperty`: Object Groups
- `addtodate`: Timing Utilities
- `airy`: Special Functions
- `all`: Finding Elements and Checking Conditions
- `allchild`: Graphics Objects
- `amd`: Mathematical Considerations
- `ancestor`: Graphics Objects
- `angle`: Complex Arithmetic
- `anova`: Tests
- `any`: Finding Elements and Checking Conditions
- `arch_fit`: Signal Processing
- `arch_rnd`: Signal Processing
- `arch_test`: Signal Processing
- `area`: Two-Dimensional Plots
- `arg`: Complex Arithmetic
- `argnames`: Inline Functions
- `argv`: Command Line Options
- `arma_rnd`: Signal Processing
- `arrayfun`: Applying a Function to an Array
- `asctime`: Timing Utilities
- `asec`: Trigonometry
- `asecd`: Trigonometry
- `asech`: Trigonometry
- `asin`: Trigonometry
- `asind`: Trigonometry
- `asinh`: Trigonometry
- `assert`: Test Functions
- `assignin`: Evaluation in a Different Context
- `atan`: Trigonometry
- `atan2`: Trigonometry
- `atand`: Trigonometry
- `atanh`: Trigonometry
- `atexit`: Quitting Octave
- `autocor`: Signal Processing
- `autocov`: Signal Processing
- `autoload`: Overloading and Autoloading
- `autoreg_matrix`: Signal Processing
- `autumn`: Representing Images
- `available_backends`: Graphics backends
- `axes`: Graphics Objects
- `axis`: Two-Dimensional Plots
- `backend`: Graphics backends
- `balance`: Basic Matrix Functions
- `bar`: Two-Dimensional Plots
- `barh`: Two-Dimensional Plots
- `bartlett`: Signal Processing
- `bartlett_test`: Tests
- `base2dec`: String Conversions
- `beep`: Raising Errors
- `beep_on_error`: Raising Errors
- `besselh`: Special Functions
- `besseli`: Special Functions
- `besselj`: Special Functions
- `besselk`: Special Functions
- `bessely`: Special Functions
- `beta`: Special Functions
- `betacdf`: Distributions
- `betainc`: Special Functions
- `betainv`: Distributions
- `betaln`: Special Functions
- `betapdf`: Distributions
- `betarnd`: Random Number Generation
- `bicgstab`: Specialized Solvers
- `bicubic`: Multi-dimensional Interpolation
- `bin2dec`: String Conversions
- `bincoeff`: Special Functions
- `binocdf`: Distributions
- `binoinv`: Distributions
- `binopdf`: Distributions
- `binornd`: Random Number Generation
- `bitand`: Bit Manipulations
- `bitcmp`: Bit Manipulations
- `bitget`: Bit Manipulations
- `bitmax`: Bit Manipulations
- `bitor`: Bit Manipulations
- `bitset`: Bit Manipulations
- `bitshift`: Bit Manipulations
- `bitxor`: Bit Manipulations
- `blackman`: Signal Processing
- `blanks`: Creating Strings
- `blkdiag`: Rearranging Matrices
- `bone`: Representing Images
- `box`: Plot Annotations
- `brighten`: Representing Images
- `bsxfun`: Applying a Function to an Array
- `bug_report`: Reporting Bugs
- `bug_report`: Bug Lists
- `builtin`: Overloading and Autoloading
- `bunzip2`: File Archiving Utilities
- `byte_size`: Matrices and Arrays in Oct-Files
- `bzip2`: File Archiving Utilities
- `C`: Status of Variables
- `calendar`: Timing Utilities
- `canonicalize_file_name`: Filesystem Utilities
- `cart2pol`: Coordinate Transformations
- `cart2sph`: Coordinate Transformations
- `cast`: Built-in Data Types
- `cat`: Rearranging Matrices
- `cauchy_cdf`: Distributions
- `cauchy_inv`: Distributions
- `cauchy_pdf`: Distributions
- `cauchy_rnd`: Random Number Generation
- `caxis`: Two-Dimensional Plots
- `ccolamd`: Mathematical Considerations
- `cd`: Current Working Directory
- `cd`: A Sample Command Description
- `ceil`: Utility Functions
- `cell`: Creating Cell Arrays
- `cell2mat`: Processing Data in Cell Arrays
- `cell2struct`: Processing Data in Cell Arrays
- `celldisp`: Basic Usage of Cell Arrays
- `cellfun`: Processing Data in Cell Arrays
- `cellidx`: Cell Arrays of Strings
- `cellstr`: Cell Arrays of Strings
- `center`: Basic Statistical Functions
- `cgs`: Specialized Solvers
- `char`: Concatenating Strings
- `chdir`: Current Working Directory
- `chdir`: A Sample Command Description
- `chi2cdf`: Distributions
- `chi2inv`: Distributions
- `chi2rnd`: Random Number Generation
- `chisquare_pdf`: Distributions
- `chisquare_test_homogeneity`: Tests
- `chisquare_test_independence`: Tests
- `chol`: Matrix Factorizations
- `chol2inv`: Matrix Factorizations
- `choldelete`: Matrix Factorizations
- `cholinsert`: Matrix Factorizations
- `cholinv`: Matrix Factorizations
- `cholshift`: Matrix Factorizations
- `cholupdate`: Matrix Factorizations
- `circshift`: Rearranging Matrices
- `cla`: Graphics Objects
- `clabel`: Plot Annotations
- `class`: Built-in Data Types
- `clc`: Cursor Motion
- `clear`: Status of Variables
- `clf`: Graphics Objects
- `clock`: Timing Utilities
- `cloglog`: Basic Statistical Functions
- `close`: Graphics Objects
- `closereq`: Graphics Objects
- `colamd`: Mathematical Considerations
- `colloc`: Orthogonal Collocation
- `colon`: Indexing Objects
- `colorbar`: Plot Annotations
- `colormap`: Representing Images
- `colperm`: Mathematical Considerations
- `columns`: Object Sizes
- `comet`: Two-Dimensional Plots
- command: Installing and Removing Packages
- `command_line_path`: Manipulating the load path
- `common_size`: Finding Elements and Checking Conditions
- `commutation_matrix`: Special Functions
- `compan`: Finding Roots
- `compass`: Two-Dimensional Plots
- `complement`: Set Operations
- `completion_append_char`: Commands For Completion
- `completion_matches`: Commands For Completion
- `complex`: Numeric Data Types
- `computer`: System Information
- `cond`: Basic Matrix Functions
- `condest`: Sparse Linear Algebra
- `confirm_recursive_rmdir`: Filesystem Utilities
- `conj`: Complex Arithmetic
- `contour`: Two-Dimensional Plots
- `contour3`: Two-Dimensional Plots
- `contourc`: Two-Dimensional Plots
- `contourf`: Two-Dimensional Plots
- `contrast`: Representing Images
- `conv`: Products of Polynomials
- `conv2`: Products of Polynomials
- `convhull`: Convex Hull
- `convhulln`: Convex Hull
- `convn`: Products of Polynomials
- `cool`: Representing Images
- `copper`: Representing Images
- `copyfile`: Filesystem Utilities
- `cor`: Descriptive Statistics
- `cor_test`: Tests
- `corrcoef`: Descriptive Statistics
- `cos`: Trigonometry
- `cosd`: Trigonometry
- `cosh`: Trigonometry
- `cot`: Trigonometry
- `cotd`: Trigonometry
- `coth`: Trigonometry
- `cov`: Descriptive Statistics
- `cplxpair`: Complex Arithmetic
- `cputime`: Timing Utilities
- `crash_dumps_octave_core`: Saving Data on Unexpected Exits
- `cross`: Utility Functions
- `csc`: Trigonometry
- `cscd`: Trigonometry
- `csch`: Trigonometry
- `cstrcat`: Concatenating Strings
- `csvread`: Simple File I/O
- `csvwrite`: Simple File I/O
- `csymamd`: Mathematical Considerations
- `ctime`: Timing Utilities
- `cummax`: Utility Functions
- `cummin`: Utility Functions
- `cumprod`: Sums and Products
- `cumsum`: Sums and Products
- `cumtrapz`: Functions of One Variable
- `cut`: Basic Statistical Functions
- `cylinder`: Three-dimensional Geometric Shapes
- `daspk`: Differential-Algebraic Equations
- `daspk_options`: Differential-Algebraic Equations
- `dasrt`: Differential-Algebraic Equations
- `dasrt_options`: Differential-Algebraic Equations
- `dassl`: Differential-Algebraic Equations
- `dassl_options`: Differential-Algebraic Equations
- `date`: Timing Utilities
- `datenum`: Timing Utilities
- `datestr`: Timing Utilities
- `datetick`: Timing Utilities
- `datevec`: Timing Utilities
- `dbclear`: Breakpoints
- `dbcont`: Leaving Debug Mode
- `dbdown`: Call Stack
- `dblquad`: Functions of Multiple Variables
- `dbquit`: Leaving Debug Mode
- `dbstack`: Call Stack
- `dbstatus`: Breakpoints
- `dbstep`: Debug Mode
- `dbstop`: Breakpoints
- `dbtype`: Debug Mode
- `dbup`: Call Stack
- `dbwhere`: Debug Mode
- `deal`: Variable-length Return Lists
- `deblank`: Manipulating Strings
- `debug_on_error`: Entering Debug Mode
- `debug_on_interrupt`: Entering Debug Mode
- `debug_on_warning`: Entering Debug Mode
- `dec2base`: String Conversions
- `dec2bin`: String Conversions
- `dec2hex`: String Conversions
- `deconv`: Products of Polynomials
- `default_save_options`: Simple File I/O
- `del2`: Utility Functions
- `delaunay`: Delaunay Triangulation
- `delaunay3`: Delaunay Triangulation
- `delaunayn`: Delaunay Triangulation
- `delete`: Graphics Objects
- `dellistener`: Object Groups
- `demo`: Demonstration Functions
- `det`: Basic Matrix Functions
- `detrend`: Signal Processing
- `diag`: Rearranging Matrices
- `diary`: Diary and Echo Commands
- `diff`: Finding Elements and Checking Conditions
- `diffpara`: Signal Processing
- `diffuse`: Three-Dimensional Plotting
- `dims`: Matrices and Arrays in Oct-Files
- `dir`: Current Working Directory
- `discrete_cdf`: Distributions
- `discrete_inv`: Distributions
- `discrete_pdf`: Distributions
- `discrete_rnd`: Random Number Generation
- `disp`: Terminal Output
- `dispatch`: Overloading and Autoloading
- `display`: Manipulating Classes
- `dlmread`: Simple File I/O
- `dlmwrite`: Simple File I/O
- `dmperm`: Mathematical Considerations
- `dmult`: Basic Matrix Functions
- `do_string_escapes`: String Conversions
- `doc_cache_file`: Getting Help
- `dos`: Controlling Subprocesses
- `dot`: Basic Matrix Functions
- `double`: Numeric Data Types
- `drawnow`: Graphics Objects
- `dsearch`: Identifying points in Triangulation
- `dsearchn`: Identifying points in Triangulation
- `dup2`: Controlling Subprocesses
- `duplication_matrix`: Special Functions
- `durbinlevinson`: Signal Processing
- `e`: Mathematical Constants
- `echo`: Diary and Echo Commands
- `echo_executing_commands`: Diary and Echo Commands
- `edit`: Function Files
- `edit_history`: Commands For History
- `EDITOR`: Commands For History
- `eig`: Basic Matrix Functions
- `elem`: Matrices and Arrays in Oct-Files
- `ellipsoid`: Three-dimensional Geometric Shapes
- `empirical_cdf`: Distributions
- `empirical_inv`: Distributions
- `empirical_pdf`: Distributions
- `empirical_rnd`: Random Number Generation
- `endgrent`: Group Database Functions
- `endpwent`: Password Database Functions
- `eomday`: Timing Utilities
- `eps`: Mathematical Constants
- `erf`: Special Functions
- `erfc`: Special Functions
- `erfinv`: Special Functions
- `errno`: Catching Errors
- `errno_list`: Catching Errors
- `error`: Raising Errors
- `errorbar`: Two-Dimensional Plots
- `etime`: Timing Utilities
- `etree`: Information
- `etreeplot`: Information
- `eval`: Evaluation
- `evalin`: Evaluation in a Different Context
- `example`: Demonstration Functions
- `exec`: Controlling Subprocesses
- `EXEC_PATH`: Controlling Subprocesses
- `exist`: Status of Variables
- `exit`: Quitting Octave
- `exp`: Exponents and Logarithms
- `expcdf`: Distributions
- `expinv`: Distributions
- `expm`: Functions of a Matrix
- `expm1`: Exponents and Logarithms
- `exppdf`: Distributions
- `exprnd`: Random Number Generation
- `eye`: Special Utility Matrices
- `ezcontour`: Two-dimensional Function Plotting
- `ezcontourf`: Two-dimensional Function Plotting
- `ezmesh`: Three-dimensional Function Plotting
- `ezmeshc`: Three-dimensional Function Plotting
- `ezplot`: Two-dimensional Function Plotting
- `ezplot3`: Three-dimensional Function Plotting
- `ezpolar`: Two-dimensional Function Plotting
- `ezsurf`: Three-dimensional Function Plotting
- `ezsurfc`: Three-dimensional Function Plotting
- `f_test_regression`: Tests
- `factor`: Utility Functions
- `factorial`: Utility Functions
- `fail`: Test Functions
- `false`: Logical Values
- `fcdf`: Distributions
- `fclear`: EOF and Errors
- `fclose`: Opening and Closing Files
- `fcntl`: Controlling Subprocesses
- `fdisp`: Simple File I/O
- `feather`: Two-Dimensional Plots
- `feof`: EOF and Errors
- `ferror`: EOF and Errors
- `feval`: Calling a Function by its Name
- `fflush`: Paging Screen Output
- `fft`: Signal Processing
- `fft2`: Signal Processing
- `fftconv`: Signal Processing
- `fftfilt`: Signal Processing
- `fftn`: Signal Processing
- `fftshift`: Signal Processing
- `fftw`: Signal Processing
- `fgetl`: Line-Oriented Input
- `fgets`: Line-Oriented Input
- field: Function Files
- `fieldnames`: Manipulating Structures
- `figure`: Multiple Plot Windows
- `file_in_loadpath`: Manipulating the load path
- `file_in_path`: Filesystem Utilities
- `fileattrib`: Filesystem Utilities
- `filemarker`: Filesystem Utilities
- `fileparts`: Filesystem Utilities
- `filesep`: Filesystem Utilities
- `fill`: Graphics Objects
- `filter`: Signal Processing
- `filter2`: Signal Processing
- `find`: Finding Elements and Checking Conditions
- `find_dir_in_path`: Manipulating the load path
- `findall`: Searching Properties
- `findobj`: Searching Properties
- `findstr`: Manipulating Strings
- `finite`: Finding Elements and Checking Conditions
- `finv`: Distributions
- `fix`: Utility Functions
- `fixed_point_format`: Matrices
- `flag`: Representing Images
- `flipdim`: Rearranging Matrices
- `fliplr`: Rearranging Matrices
- `flipud`: Rearranging Matrices
- `floor`: Utility Functions
- `fmod`: Utility Functions
- `fnmatch`: Filesystem Utilities
- `foo`: A Sample Function Description
- `fopen`: Opening and Closing Files
- `fork`: Controlling Subprocesses
- `format`: Terminal Output
- `formula`: Inline Functions
- `fortran_vec`: Matrices and Arrays in Oct-Files
- `fpdf`: Distributions
- `fplot`: Two-dimensional Function Plotting
- `fprintf`: Formatted Output
- `fputs`: Simple Output
- `fractdiff`: Signal Processing
- `fread`: Binary I/O
- `freport`: EOF and Errors
- `freqz`: Signal Processing
- `freqz_plot`: Signal Processing
- `frewind`: File Positioning
- `frnd`: Random Number Generation
- `fscanf`: Formatted Input
- `fseek`: File Positioning
- `fsolve`: Nonlinear Equations
- `fstat`: Filesystem Utilities
- `ftell`: File Positioning
- `full`: Creating Sparse Matrices
- `fullfile`: Filesystem Utilities
- `func2str`: Function Handles
- function\_name: Getting Help
- `functions`: Function Handles
- `fwrite`: Binary I/O
- `fzero`: Nonlinear Equations
- `gamcdf`: Distributions
- `gaminv`: Distributions
- `gamma`: Special Functions
- `gammainc`: Special Functions
- `gammaln`: Special Functions
- `gampdf`: Distributions
- `gamrnd`: Random Number Generation
- `gca`: Graphics Objects
- `gcbf`: Callbacks
- `gcbo`: Callbacks
- `gcd`: Utility Functions
- `gcf`: Graphics Objects
- `genpath`: Manipulating the load path
- `genvarname`: Variables
- `geocdf`: Distributions
- `geoinv`: Distributions
- `geopdf`: Distributions
- `geornd`: Random Number Generation
- `get`: Graphics Objects
- `getegid`: Process ID Information
- `getenv`: Environment Variables
- `geteuid`: Process ID Information
- `getfield`: Manipulating Structures
- `getgid`: Process ID Information
- `getgrent`: Group Database Functions
- `getgrgid`: Group Database Functions
- `getgrnam`: Group Database Functions
- `getpgrp`: Process ID Information
- `getpid`: Process ID Information
- `getppid`: Process ID Information
- `getpwent`: Password Database Functions
- `getpwnam`: Password Database Functions
- `getpwuid`: Password Database Functions
- `getrusage`: System Information
- `getuid`: Process ID Information
- `ginput`: Interacting with plots
- `givens`: Basic Matrix Functions
- `glob`: Filesystem Utilities
- `glpk`: Linear Programming
- `gls`: Linear Least Squares
- `gmap40`: Representing Images
- `gmtime`: Timing Utilities
- `gnuplot_binary`: Interaction with gnuplot
- `gplot`: Information
- `gradient`: Utility Functions
- `gray`: Representing Images
- `gray2ind`: Representing Images
- `grid`: Plot Annotations
- `griddata`: Interpolation on Scattered Data
- `griddata3`: Interpolation on Scattered Data
- `griddatan`: Interpolation on Scattered Data
- `gtext`: Interacting with plots
- `gunzip`: File Archiving Utilities
- `gzip`: File Archiving Utilities
- `hadamard`: Famous Matrices
- `hamming`: Signal Processing
- `hankel`: Famous Matrices
- `hanning`: Signal Processing
- `hess`: Matrix Factorizations
- `hex2dec`: String Conversions
- `hex2num`: String Conversions
- `hggroup`: Object Groups
- `hidden`: Three-Dimensional Plotting
- `hilb`: Famous Matrices
- `hist`: Two-Dimensional Plots
- `histc`: Basic Statistical Functions
- `history`: Commands For History
- `history_file`: Commands For History
- `history_size`: Commands For History
- `history_timestamp_format_string`: Commands For History
- `hold`: Graphics Objects
- `home`: Cursor Motion
- `horzcat`: Rearranging Matrices
- `hot`: Representing Images
- `hotelling_test`: Tests
- `hotelling_test_2`: Tests
- `housh`: Matrix Factorizations
- `hsv`: Representing Images
- `hsv2rgb`: Color Conversion
- `hurst`: Signal Processing
- `hygecdf`: Distributions
- `hygeinv`: Distributions
- `hygepdf`: Distributions
- `hygernd`: Random Number Generation
- `hypot`: Utility Functions
- `I`: Mathematical Constants
- `idivide`: Integer Arithmetic
- `ifft`: Signal Processing
- `ifftn`: Signal Processing
- `ifftshift`: Signal Processing
- `ignore_function_time_stamp`: Function Files
- `imag`: Complex Arithmetic
- `image`: Displaying Images
- `IMAGE_PATH`: Loading and Saving Images
- `image_viewer`: Displaying Images
- `imagesc`: Displaying Images
- `imfinfo`: Loading and Saving Images
- `imread`: Loading and Saving Images
- `imshow`: Displaying Images
- `imwrite`: Loading and Saving Images
- `ind2gray`: Representing Images
- `ind2rgb`: Representing Images
- `ind2sub`: Index Expressions
- `index`: Manipulating Strings
- `Inf`: Mathematical Constants
- `inferiorto`: Precedence of Objects
- `info`: Getting Help
- `info_file`: Getting Help
- `info_program`: Getting Help
- `inline`: Inline Functions
- `inpolygon`: Voronoi Diagrams
- `input`: Terminal Input
- `inputname`: Defining Functions
- `int16`: Integer Data Types
- `int2str`: Conversion of Numerical Data to Strings
- `int32`: Integer Data Types
- `int64`: Integer Data Types
- `int8`: Integer Data Types
- `interp1`: One-dimensional Interpolation
- `interp1q`: One-dimensional Interpolation
- `interp2`: Multi-dimensional Interpolation
- `interp3`: Multi-dimensional Interpolation
- `interpft`: One-dimensional Interpolation
- `interpn`: Multi-dimensional Interpolation
- `intersect`: Set Operations
- `intmax`: Integer Data Types
- `intmin`: Integer Data Types
- `intwarning`: Integer Data Types
- `inv`: Basic Matrix Functions
- `inverse`: Basic Matrix Functions
- `invhilb`: Famous Matrices
- `ipermute`: Rearranging Matrices
- `iqr`: Basic Statistical Functions
- `is_absolute_filename`: Filesystem Utilities
- `is_duplicate_entry`: Finding Elements and Checking Conditions
- `is_leap_year`: Timing Utilities
- `is_rooted_relative_filename`: Filesystem Utilities
- `isa`: Built-in Data Types
- `isalnum`: Character Class Functions
- `isalpha`: Character Class Functions
- `isascii`: Character Class Functions
- `iscell`: Basic Usage of Cell Arrays
- `iscellstr`: Cell Arrays of Strings
- `ischar`: Character Arrays
- `iscntrl`: Character Class Functions
- `iscommand`: Commands
- `iscomplex`: Predicates for Numeric Objects
- `isdebugmode`: Debug Mode
- `isdefinite`: Predicates for Numeric Objects
- `isdigit`: Character Class Functions
- `isdir`: Filesystem Utilities
- `isempty`: Object Sizes
- `isequal`: Comparison Ops
- `isequalwithequalnans`: Comparison Ops
- `isfield`: Manipulating Structures
- `isfigure`: Graphics Objects
- `isfloat`: Predicates for Numeric Objects
- `isglobal`: Global Variables
- `isgraph`: Character Class Functions
- `ishandle`: Graphics Objects
- `ishghandle`: Graphics Objects
- `ishold`: Graphics Objects
- `isieee`: System Information
- `isinf`: Finding Elements and Checking Conditions
- `isinteger`: Integer Data Types
- `isletter`: Character Class Functions
- `islogical`: Predicates for Numeric Objects
- `islower`: Character Class Functions
- `ismac`: System Information
- `ismatrix`: Predicates for Numeric Objects
- `ismember`: Set Operations
- `ismethod`: Creating a Class
- `isna`: Missing Data
- `isnan`: Finding Elements and Checking Conditions
- `isnull`: Object Sizes
- `isnumeric`: Predicates for Numeric Objects
- `isobject`: Creating a Class
- `ispc`: System Information
- `isprime`: Predicates for Numeric Objects
- `isprint`: Character Class Functions
- `ispunct`: Character Class Functions
- `israwcommand`: Commands
- `isreal`: Predicates for Numeric Objects
- `isscalar`: Predicates for Numeric Objects
- `issorted`: Rearranging Matrices
- `isspace`: Character Class Functions
- `issparse`: Information
- `issquare`: Predicates for Numeric Objects
- `isstrprop`: Character Class Functions
- `isstruct`: Creating Structures
- `issymmetric`: Predicates for Numeric Objects
- `isunix`: System Information
- `isupper`: Character Class Functions
- `isvarname`: Variables
- `isvector`: Predicates for Numeric Objects
- `isxdigit`: Character Class Functions
- `jet`: Representing Images
- `kbhit`: Terminal Input
- `kendall`: Basic Statistical Functions
- `keyboard`: Breakpoints
- `kill`: Controlling Subprocesses
- `kolmogorov_smirnov_cdf`: Distributions
- `kolmogorov_smirnov_test`: Tests
- `kolmogorov_smirnov_test_2`: Tests
- `kron`: Functions of a Matrix
- `kruskal_wallis_test`: Tests
- `krylov`: Matrix Factorizations
- `kurtosis`: Descriptive Statistics
- `laplace_cdf`: Distributions
- `laplace_inv`: Distributions
- `laplace_pdf`: Distributions
- `laplace_rnd`: Random Number Generation
- `lasterr`: Catching Errors
- `lasterror`: Catching Errors
- `lastwarn`: Issuing Warnings
- `lcm`: Utility Functions
- `legend`: Plot Annotations
- `legendre`: Special Functions
- `length`: Object Sizes
- `lgamma`: Special Functions
- `license`: System Information
- `lin2mu`: Audio Processing
- `line`: Graphics Objects
- `link`: Filesystem Utilities
- `linkprop`: Object Groups
- `linspace`: Special Utility Matrices
- `list_primes`: Utility Functions
- `load`: Simple File I/O
- `loadaudio`: Audio Processing
- `loadobj`: Manipulating Classes
- `localtime`: Timing Utilities
- `log`: Exponents and Logarithms
- `log10`: Exponents and Logarithms
- `log1p`: Exponents and Logarithms
- `log2`: Exponents and Logarithms
- `logical`: Logical Values
- `logistic_cdf`: Distributions
- `logistic_inv`: Distributions
- `logistic_pdf`: Distributions
- `logistic_regression`: Models
- `logistic_rnd`: Random Number Generation
- `logit`: Basic Statistical Functions
- `loglog`: Two-Dimensional Plots
- `loglogerr`: Two-Dimensional Plots
- `logm`: Functions of a Matrix
- `logncdf`: Distributions
- `logninv`: Distributions
- `lognpdf`: Distributions
- `lognrnd`: Random Number Generation
- `logspace`: Special Utility Matrices
- `lookfor`: Getting Help
- `lookup`: One-dimensional Interpolation
- `lower`: String Conversions
- `ls`: Current Working Directory
- `ls_command`: Current Working Directory
- `lsode`: Ordinary Differential Equations
- `lsode_options`: Ordinary Differential Equations
- `lsqnonneg`: Linear Least Squares
- `lstat`: Filesystem Utilities
- `lu`: Matrix Factorizations
- `luinc`: Iterative Techniques
- `magic`: Famous Matrices
- `mahalanobis`: Basic Statistical Functions
- `make_absolute_filename`: Filesystem Utilities
- `makeinfo_program`: Getting Help
- `manova`: Tests
- `mark_as_command`: Commands
- `mark_as_rawcommand`: Commands
- `mat2cell`: Creating Cell Arrays
- `mat2str`: Conversion of Numerical Data to Strings
- `matrix_type`: Basic Matrix Functions
- `max`: Utility Functions
- `max_recursion_depth`: Recursion
- `mcnemar_test`: Tests
- `md5sum`: Hashing Functions
- `mean`: Descriptive Statistics
- `meansq`: Descriptive Statistics
- `median`: Descriptive Statistics
- `menu`: Terminal Input
- `mesh`: Three-Dimensional Plotting
- `meshc`: Three-Dimensional Plotting
- `meshgrid`: Three-Dimensional Plotting
- `meshz`: Three-Dimensional Plotting
- `methods`: Creating a Class
- `mex`: Getting Started with Mex-Files
- `mexext`: Getting Started with Mex-Files
- `mfilename`: Function Files
- `min`: Utility Functions
- `mislocked`: Function Locking
- `mkdir`: Filesystem Utilities
- `mkfifo`: Filesystem Utilities
- `mkoctfile`: Getting Started with Oct-Files
- `mkpp`: Polynomial Interpolation
- `mkstemp`: Temporary Files
- `mktime`: Timing Utilities
- `mlock`: Function Locking
- `mod`: Utility Functions
- `mode`: Descriptive Statistics
- `moment`: Descriptive Statistics
- `more`: Paging Screen Output
- `movefile`: Filesystem Utilities
- `mpoles`: Finding Roots
- `mu2lin`: Audio Processing
- `munlock`: Function Locking
- `NA`: Missing Data
- name: Getting Help
- name: Function Files
- `namelengthmax`: Variables
- `NaN`: Mathematical Constants
- `nargchk`: Multiple Return Values
- `nargin`: Defining Functions
- `nargout`: Multiple Return Values
- `nargoutchk`: Multiple Return Values
- `native_float_format`: Simple File I/O
- `nbincdf`: Distributions
- `nbininv`: Distributions
- `nbinpdf`: Distributions
- `nbinrnd`: Random Number Generation
- `nchoosek`: Basic Statistical Functions
- `ndgrid`: Three-Dimensional Plotting
- `ndims`: Object Sizes
- `nelem`: Matrices and Arrays in Oct-Files
- `newplot`: Graphics Objects
- `news`: Getting Help
- `newtroot`: Calling a Function by its Name
- `nextpow2`: Exponents and Logarithms
- `nnz`: Information
- `nonzeros`: Information
- `norm`: Basic Matrix Functions
- `normcdf`: Distributions
- `normest`: Sparse Linear Algebra
- `norminv`: Distributions
- `normpdf`: Distributions
- `normrnd`: Random Number Generation
- `now`: Timing Utilities
- `nthroot`: Exponents and Logarithms
- `ntsc2rgb`: Color Conversion
- `null`: Basic Matrix Functions
- `num2cell`: Creating Cell Arrays
- `num2hex`: String Conversions
- `num2str`: Conversion of Numerical Data to Strings
- `numel`: Object Sizes
- `nzmax`: Information
- `ocean`: Representing Images
- `octave_config_info`: System Information
- `octave_core_file_limit`: Saving Data on Unexpected Exits
- `octave_core_file_name`: Saving Data on Unexpected Exits
- `octave_core_file_options`: Saving Data on Unexpected Exits
- `OCTAVE_HOME`: System Information
- `OCTAVE_VERSION`: System Information
- `ols`: Linear Least Squares
- `onenormest`: Sparse Linear Algebra
- `ones`: Special Utility Matrices
- `operator ()`: Matrices and Arrays in Oct-Files
- `optimget`: Linear Least Squares
- `optimset`: Linear Least Squares
- `options`: Status of Variables
- `orderfields`: Manipulating Structures
- `orient`: Printing Plots
- `orth`: Basic Matrix Functions
- `output_max_field_width`: Matrices
- `output_precision`: Matrices
- `P_tmpdir`: Filesystem Utilities
- `pack`: File Archiving Utilities
- `page_output_immediately`: Paging Screen Output
- `page_screen_output`: Paging Screen Output
- `PAGER`: Paging Screen Output
- `PAGER_FLAGS`: Paging Screen Output
- `pareto`: Two-Dimensional Plots
- `parseparams`: Variable-length Argument Lists
- `pascal`: Famous Matrices
- `patch`: Graphics Objects
- `path`: Manipulating the load path
- `pathdef`: Manipulating the load path
- `pathsep`: Manipulating the load path
- `pause`: Timing Utilities
- `pcg`: Iterative Techniques
- `pchip`: Signal Processing
- `pclose`: Controlling Subprocesses
- `pcolor`: Two-Dimensional Plots
- `pcr`: Iterative Techniques
- `peaks`: Test Plotting Functions
- `periodogram`: Signal Processing
- `perl`: Controlling Subprocesses
- `perms`: Basic Statistical Functions
- `permute`: Rearranging Matrices
- `pi`: Mathematical Constants
- `pie`: Two-Dimensional Plots
- `pink`: Representing Images
- `pinv`: Basic Matrix Functions
- `pipe`: Controlling Subprocesses
- `pkg`: Packages
- `planerot`: Basic Matrix Functions
- `playaudio`: Audio Processing
- `plot`: Two-Dimensional Plots
- `plot3`: Three-Dimensional Plotting
- `plotmatrix`: Two-Dimensional Plots
- `plotyy`: Two-Dimensional Plots
- `poisscdf`: Distributions
- `poissinv`: Distributions
- `poisspdf`: Distributions
- `poissrnd`: Random Number Generation
- `pol2cart`: Coordinate Transformations
- `polar`: Two-Dimensional Plots
- `poly`: Miscellaneous Functions
- `polyarea`: Voronoi Diagrams
- `polyder`: Derivatives and Integrals
- `polyderiv`: Derivatives and Integrals
- `polyfit`: Polynomial Interpolation
- `polygcd`: Products of Polynomials
- `polyint`: Derivatives and Integrals
- `polyinteg`: Derivatives and Integrals
- `polyout`: Miscellaneous Functions
- `polyreduce`: Miscellaneous Functions
- `polyval`: Evaluating Polynomials
- `polyvalm`: Evaluating Polynomials
- `popen`: Controlling Subprocesses
- `popen2`: Controlling Subprocesses
- `pow2`: Exponents and Logarithms
- `ppplot`: Statistical Plots
- `ppval`: Polynomial Interpolation
- `prctile`: Descriptive Statistics
- `prepad`: Rearranging Matrices
- `primes`: Utility Functions
- `print`: Printing Plots
- `print_empty_dimensions`: Empty Matrices
- `print_usage`: Raising Errors
- `printf`: Formatted Output
- `prism`: Representing Images
- `probit`: Basic Statistical Functions
- `prod`: Sums and Products
- `program_name`: Command Line Options
- `prop_test_2`: Tests
- `PS1`: Customizing the Prompt
- `PS2`: Customizing the Prompt
- `PS4`: Customizing the Prompt
- `putenv`: Environment Variables
- `puts`: Simple Output
- `pwd`: Current Working Directory
- `qp`: Quadratic Programming
- `qqplot`: Statistical Plots
- `qr`: Matrix Factorizations
- `qrdelete`: Matrix Factorizations
- `qrinsert`: Matrix Factorizations
- `qrshift`: Matrix Factorizations
- `qrupdate`: Matrix Factorizations
- `quad`: Functions of One Variable
- `quad_options`: Functions of One Variable
- `quadgk`: Functions of One Variable
- `quadl`: Functions of One Variable
- `quadv`: Functions of One Variable
- `quantile`: Descriptive Statistics
- `quit`: Quitting Octave
- `quiver`: Two-Dimensional Plots
- `quiver3`: Two-Dimensional Plots
- `qz`: Matrix Factorizations
- `qzhess`: Matrix Factorizations
- `rainbow`: Representing Images
- `rand`: Special Utility Matrices
- `rande`: Special Utility Matrices
- `randg`: Special Utility Matrices
- `randn`: Special Utility Matrices
- `randp`: Special Utility Matrices
- `randperm`: Special Utility Matrices
- `range`: Basic Statistical Functions
- `rank`: Basic Matrix Functions
- `ranks`: Basic Statistical Functions
- `rat`: Rational Approximations
- `rats`: Rational Approximations
- `rcond`: Basic Matrix Functions
- `re_read_readline_init_file`: Customizing readline
- `read_readline_init_file`: Customizing readline
- `readdir`: Filesystem Utilities
- `readlink`: Filesystem Utilities
- `real`: Complex Arithmetic
- `reallog`: Exponents and Logarithms
- `realmax`: Mathematical Constants
- `realmin`: Mathematical Constants
- `realpow`: Exponents and Logarithms
- `realsqrt`: Exponents and Logarithms
- `record`: Audio Processing
- `rectangle_lw`: Signal Processing
- `rectangle_sw`: Signal Processing
- `rectint`: Voronoi Diagrams
- `refresh`: Graphics Objects
- `refreshdata`: Data sources in object groups
- `regexp`: Manipulating Strings
- `regexpi`: Manipulating Strings
- `regexprep`: Manipulating Strings
- `regexptranslate`: Manipulating Strings
- `rehash`: Manipulating the load path
- `rem`: Utility Functions
- `rename`: Filesystem Utilities
- `repmat`: Special Utility Matrices
- `reshape`: Rearranging Matrices
- `residue`: Products of Polynomials
- `resize`: Rearranging Matrices
- `resize`: Matrices and Arrays in Oct-Files
- `resize`: Rearranging Matrices
- `restoredefaultpath`: Manipulating the load path
- `rethrow`: Catching Errors
- `return`: Returning From a Function
- `rgb2hsv`: Color Conversion
- `rgb2ind`: Representing Images
- `rgb2ntsc`: Color Conversion
- `ribbon`: Three-Dimensional Plotting
- `rindex`: Manipulating Strings
- `rmdir`: Filesystem Utilities
- `rmfield`: Manipulating Structures
- `rmpath`: Manipulating the load path
- `roots`: Finding Roots
- `rose`: Two-Dimensional Plots
- `rosser`: Famous Matrices
- `rot90`: Rearranging Matrices
- `rotdim`: Rearranging Matrices
- `round`: Utility Functions
- `roundb`: Utility Functions
- `rows`: Object Sizes
- `rref`: Basic Matrix Functions
- `run`: Calling a Function by its Name
- `run_count`: Basic Statistical Functions
- `run_history`: Commands For History
- `run_test`: Tests
- `rundemos`: Demonstration Functions
- `S`: Simple File I/O
- `S`: Status of Variables
- `save`: Simple File I/O
- `save_header_format_string`: Simple File I/O
- `save_precision`: Simple File I/O
- `saveaudio`: Audio Processing
- `saveobj`: Manipulating Classes
- `savepath`: Manipulating the load path
- `saving_history`: Commands For History
- `scanf`: Formatted Input
- `scatter`: Two-Dimensional Plots
- `scatter3`: Two-Dimensional Plots
- `schur`: Matrix Factorizations
- `sec`: Trigonometry
- `secd`: Trigonometry
- `sech`: Trigonometry
- `SEEK_CUR`: File Positioning
- `SEEK_END`: File Positioning
- `SEEK_SET`: File Positioning
- `semilogx`: Two-Dimensional Plots
- `semilogxerr`: Two-Dimensional Plots
- `semilogy`: Two-Dimensional Plots
- `semilogyerr`: Two-Dimensional Plots
- `set`: Graphics Objects
- `setaudio`: Audio Processing
- `setdiff`: Set Operations
- `setenv`: Environment Variables
- `setfield`: Manipulating Structures
- `setgrent`: Group Database Functions
- `setpwent`: Password Database Functions
- `setxor`: Set Operations
- `shading`: Three-Dimensional Plotting
- `shg`: Graphics Objects
- `shift`: Rearranging Matrices
- `shiftdim`: Rearranging Matrices
- `SIG`: Controlling Subprocesses
- `sighup_dumps_octave_core`: Saving Data on Unexpected Exits
- `sign`: Utility Functions
- `sign_test`: Tests
- `sigterm_dumps_octave_core`: Saving Data on Unexpected Exits
- `silent_functions`: Defining Functions
- `sin`: Trigonometry
- `sinc`: Signal Processing
- `sind`: Trigonometry
- `sinetone`: Signal Processing
- `sinewave`: Signal Processing
- `single`: Single Precision Data Types
- `sinh`: Trigonometry
- `size`: Object Sizes
- `size_equal`: Object Sizes
- `sizeof`: Object Sizes
- `skewness`: Descriptive Statistics
- `sleep`: Timing Utilities
- `slice`: Three-Dimensional Plotting
- `sombrero`: Test Plotting Functions
- `sort`: Rearranging Matrices
- `sortrows`: Rearranging Matrices
- `source`: Script Files
- `spalloc`: Creating Sparse Matrices
- `sparse`: Creating Sparse Matrices
- `sparse_auto_mutate`: Return Types of Operators and Functions
- `spaugment`: Sparse Linear Algebra
- `spconvert`: Creating Sparse Matrices
- `spdiags`: Creating Sparse Matrices
- `spearman`: Basic Statistical Functions
- `spectral_adf`: Signal Processing
- `spectral_xdf`: Signal Processing
- `specular`: Three-Dimensional Plotting
- `speed`: Demonstration Functions
- `spencer`: Signal Processing
- `speye`: Creating Sparse Matrices
- `spfun`: Creating Sparse Matrices
- `sph2cart`: Coordinate Transformations
- `sphere`: Three-dimensional Geometric Shapes
- `spinmap`: Representing Images
- `spline`: One-dimensional Interpolation
- `split_long_rows`: Matrices
- `spmax`: Creating Sparse Matrices
- `spmin`: Creating Sparse Matrices
- `spones`: Creating Sparse Matrices
- `spparms`: Sparse Linear Algebra
- `sprand`: Creating Sparse Matrices
- `sprandn`: Creating Sparse Matrices
- `sprandsym`: Creating Sparse Matrices
- `sprank`: Sparse Linear Algebra
- `spring`: Representing Images
- `sprintf`: Formatted Output
- `spstats`: Information
- `spy`: Information
- `sqp`: Nonlinear Programming
- `sqrt`: Exponents and Logarithms
- `sqrtm`: Functions of a Matrix
- `squeeze`: Object Sizes
- `sscanf`: Formatted Input
- `stairs`: Two-Dimensional Plots
- `stat`: Filesystem Utilities
- `statistics`: Descriptive Statistics
- `std`: Descriptive Statistics
- `stderr`: C-Style I/O Functions
- `stdin`: C-Style I/O Functions
- `stdout`: C-Style I/O Functions
- `stem`: Two-Dimensional Plots
- `stem3`: Two-Dimensional Plots
- `stft`: Signal Processing
- str: Getting Help
- `str2double`: String Conversions
- `str2func`: Function Handles
- `str2num`: String Conversions
- `strcat`: Concatenating Strings
- `strchr`: Manipulating Strings
- `strcmp`: Comparing Strings
- `strcmpi`: Comparing Strings
- `strfind`: Manipulating Strings
- `strftime`: Timing Utilities
- `string_fill_char`: Character Arrays
- `strjust`: String Conversions
- `strmatch`: Manipulating Strings
- `strncmp`: Comparing Strings
- `strncmpi`: Comparing Strings
- `strptime`: Timing Utilities
- `strrep`: Manipulating Strings
- `strsplit`: Manipulating Strings
- `strtok`: Manipulating Strings
- `strtrim`: Manipulating Strings
- `strtrunc`: Manipulating Strings
- `struct`: Creating Structures
- `struct2cell`: Processing Data in Structures
- `struct_levels_to_print`: Basic Usage and Examples
- `structfun`: Processing Data in Structures
- `strvcat`: Concatenating Strings
- `studentize`: Basic Statistical Functions
- `sub2ind`: Index Expressions
- `subplot`: Multiple Plots on One Page
- `subsasgn`: Indexing Objects
- `subsindex`: Indexing Objects
- `subspace`: Matrix Factorizations
- `subsref`: Indexing Objects
- `substr`: Manipulating Strings
- `substruct`: Manipulating Structures
- `sum`: Sums and Products
- `summer`: Representing Images
- `sumsq`: Sums and Products
- `superiorto`: Precedence of Objects
- `suppress_verbose_help_message`: Getting Help
- `surf`: Three-Dimensional Plotting
- `surface`: Graphics Objects
- `surfc`: Three-Dimensional Plotting
- `surfl`: Three-Dimensional Plotting
- `surfnorm`: Three-Dimensional Plotting
- `svd`: Matrix Factorizations
- `svds`: Sparse Linear Algebra
- `swapbytes`: Built-in Data Types
- `syl`: Functions of a Matrix
- `sylvester_matrix`: Famous Matrices
- `symamd`: Mathematical Considerations
- `symbfact`: Sparse Linear Algebra
- `symlink`: Filesystem Utilities
- `symrcm`: Mathematical Considerations
- `symvar`: Inline Functions
- `synthesis`: Signal Processing
- `system`: Controlling Subprocesses
- `t_test`: Tests
- `t_test_2`: Tests
- `t_test_regression`: Tests
- `table`: Basic Statistical Functions
- `tan`: Trigonometry
- `tand`: Trigonometry
- `tanh`: Trigonometry
- `tar`: File Archiving Utilities
- `tcdf`: Distributions
- `tempdir`: Filesystem Utilities
- `tempname`: Filesystem Utilities
- `test`: Test Functions
- `text`: Plot Annotations
- `tic`: Timing Utilities
- `tilde_expand`: Filesystem Utilities
- `time`: Timing Utilities
- `tinv`: Distributions
- `title`: Plot Annotations
- `tmpfile`: Temporary Files
- `tmpnam`: Temporary Files
- `toascii`: String Conversions
- `toc`: Timing Utilities
- `toeplitz`: Famous Matrices
- `tolower`: String Conversions
- `toupper`: String Conversions
- `tpdf`: Distributions
- `trace`: Basic Matrix Functions
- `trapz`: Functions of One Variable
- `treelayout`: Information
- `treeplot`: Information
- `triangle_lw`: Signal Processing
- `triangle_sw`: Signal Processing
- `tril`: Rearranging Matrices
- `trimesh`: Plotting the Triangulation
- `triplequad`: Functions of Multiple Variables
- `triplot`: Plotting the Triangulation
- `triu`: Rearranging Matrices
- `trnd`: Random Number Generation
- `true`: Logical Values
- `tsearch`: Identifying points in Triangulation
- `tsearchn`: Identifying points in Triangulation
- `typecast`: Built-in Data Types
- `typeinfo`: Data Types
- `u_test`: Tests
- `uint16`: Integer Data Types
- `uint32`: Integer Data Types
- `uint64`: Integer Data Types
- `uint8`: Integer Data Types
- `umask`: Filesystem Utilities
- `uname`: System Information
- `undo_string_escapes`: String Conversions
- `unidcdf`: Distributions
- `unidinv`: Distributions
- `unidpdf`: Distributions
- `unidrnd`: Random Number Generation
- `unifcdf`: Distributions
- `unifinv`: Distributions
- `unifpdf`: Distributions
- `unifrnd`: Random Number Generation
- `union`: Set Operations
- `unique`: Sets
- `unix`: Controlling Subprocesses
- `unlink`: Filesystem Utilities
- `unmark_command`: Commands
- `unmark_rawcommand`: Commands
- `unmkpp`: Polynomial Interpolation
- `unpack`: File Archiving Utilities
- `untar`: File Archiving Utilities
- `unwrap`: Signal Processing
- `unzip`: File Archiving Utilities
- `upper`: String Conversions
- `urlread`: Networking Utilities
- `urlwrite`: Networking Utilities
- `usage`: Raising Errors
- `usleep`: Timing Utilities
- `validatestring`: Comparing Strings
- `values`: Basic Statistical Functions
- `vander`: Famous Matrices
- `var`: Descriptive Statistics
- `var_test`: Tests
- `vec`: Rearranging Matrices
- `vech`: Rearranging Matrices
- `vectorize`: Inline Functions
- `ver`: System Information
- `version`: System Information
- `vertcat`: Rearranging Matrices
- `view`: Three-Dimensional Plotting
- `voronoi`: Voronoi Diagrams
- `voronoin`: Voronoi Diagrams
- `waitforbuttonpress`: Interacting with plots
- `waitpid`: Controlling Subprocesses
- `warning`: Issuing Warnings
- `warranty`: Getting Help
- `wavread`: Audio Processing
- `wavwrite`: Audio Processing
- `wblcdf`: Distributions
- `wblinv`: Distributions
- `wblpdf`: Distributions
- `wblrnd`: Random Number Generation
- `WCOREDUMP`: Controlling Subprocesses
- `weekday`: Timing Utilities
- `welch_test`: Tests
- `WEXITSTATUS`: Controlling Subprocesses
- `what`: Status of Variables
- `which`: Status of Variables
- `white`: Representing Images
- `who`: Status of Variables
- `whos`: Status of Variables
- `whos_line_format`: Status of Variables
- `wienrnd`: Random Number Generation
- `WIFCONTINUED`: Controlling Subprocesses
- `WIFEXITED`: Controlling Subprocesses
- `WIFSIGNALED`: Controlling Subprocesses
- `WIFSTOPPED`: Controlling Subprocesses
- `wilcoxon_test`: Tests
- `wilkinson`: Famous Matrices
- `winter`: Representing Images
- `WNOHANG`: Controlling Subprocesses
- `WSTOPSIG`: Controlling Subprocesses
- `WTERMSIG`: Controlling Subprocesses
- `WUNTRACED`: Controlling Subprocesses
- `xlabel`: Plot Annotations
- `xlim`: Two-Dimensional Plots
- `xor`: Finding Elements and Checking Conditions
- `yes_or_no`: Terminal Input
- `ylabel`: Plot Annotations
- `yulewalker`: Signal Processing
- `z_test`: Tests
- `z_test_2`: Tests
- `zeros`: Special Utility Matrices
- `zip`: File Archiving Utilities
- `zlabel`: Plot Annotations
